# Enhanced human adipose‐derived stem cells with VEGFA and bFGF mRNA promote stable vascular regeneration and improve cardiac function following myocardial infarction

**DOI:** 10.1002/ctm2.70250

**Published:** 2025-02-26

**Authors:** Kaixiang Li, Runjiao Luo, Xindi Yu, Wei Dong, Guoliang Hao, Dan Hu, Ziyou Yu, Minglu Liu, Tingting Lu, Xiangying Wang, Xin Tang, Xinjun Lin, Huijing Wang, Wei Wang, Wei Fu

**Affiliations:** ^1^ Department of Pediatric Cardiothoracic Surgery Shanghai Children's Medical Center School of Medicine Shanghai Jiao Tong University Shanghai China; ^2^ Institute of Electrophysiology Henan Academy of Innovations in Medical Science Zhengzhou China; ^3^ Henan Key Laboratory of Cardiac Electrophysiology Henan SCOPE Research Institute of Electrophysiology Co. Ltd. Kaifeng China; ^4^ School of Mathematical Sciences Institute of Natural Sciences and MOE‐LSC Shanghai Jiao Tong University Shanghai China; ^5^ Department of Plastic and Reconstructive Surgery Shanghai Ninth People's Hospital School of Medicine Shanghai Jiao Tong University Shanghai China; ^6^ Institute of Pediatric Translational Medicine Shanghai Institute of Pediatric Congenital Heart Disease Shanghai Children's Medical Center School of Medicine Shanghai Jiao Tong University Shanghai China; ^7^ Basic Medical College of Bengbu Medical University Bengbu China; ^8^ Hainan Branch Shanghai Children's Medical Center School of Medicine Shanghai Jiao Tong University Sanya China; ^9^ Shanghai Key Laboratory of Tissue Engineering Shanghai 9th People's Hospital School of Medicine Shanghai Jiao Tong University Shanghai China

**Keywords:** bFGF, cell therapy, human adipose‐derived stem cell, modRNA, myocardial infarction, VEGF

## Abstract

Mesenchymal stem cell therapy involves the secretion of various factors to regulate the local microenvironment in various of diseases. This therapy offers hope for treating acute myocardial infarction (MI), which poses a serious threat to human health. However, challenges such as low paracrine efficiency and poor cell survival persist due to the harsh post‐infarction conditions, such as hypoxia. Recently, enhanced cell therapy, in which vascular endothelial growth factor A (VEGFA) and basic fibroblast growth factor (bFGF) are used as therapeutic agents to limit myocardial injury and simultaneously induce neovascularisation, has been recognised as a promising new strategy to improve the efficacy of cell therapy. Chemically synthetic modified messenger RNA (modRNA), a novel protein expression technology, enables safe, rapid, efficient and pulsatile expression of target proteins in vivo and in vitro settings. It has been widely applied in the fields of vaccine research and tissue regeneration. In this study, human adipose‐derived stem cells (hADSCs) were transfected with VEGFA and bFGF modRNA to transiently overexpress these proteins before transplantation. This modification enhanced the paracrine effect of transplanted hADSCs and promoted stability in the vascular network at the transplantation site. Overexpression of VEGFA and bFGF in hADSCs not only inhibited apoptosis but also reduced ventricular remodelling and improved cardiac function and left ventricular conduction. Overall, the additive effects of VEGFA modRNA, bFGF modRNA and hADSCs hold promise for comprehensive cardiac repair post‐MI and show substantial potential for treating ischemic heart diseases.

**Key points:**

- ModRNAs‐transfected hADSCs exhibit pulsed and transient expression, enabling efficient production of functional VEGFA and bFGF proteins.
- Intracardiac injection of these engineered hADSCs leads to the enhancement of cardiac function and the improvement of electrical conduction.
- The hADSCs^dual^ mainly exerts its effect on myocardial infarction by promoting stable vascular regeneration and suppressing cell apoptosis.

## Graphical abstract

1. ModRNAs‐transfected hADSCs exhibit pulsed and transient expression, enabling efficient production of functional VEGFA and bFGF proteins.
2. Intracardiac injection of these engineered hADSCs leads to the enhancement of cardiac function and the improvement of electrical conduction.
3. The hADSCs^dual^ mainly exerts its effect on myocardial infarction by promoting stable vascular regeneration and suppressing cell apoptosis.

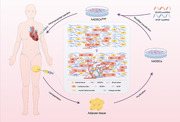

## 1 INTRODUCTION

Myocardial infarction (MI), commonly known as heart attack, is a serious condition that causes scar formation and permanent damage to myocardial cells. This damage impairs cardiac function and often leads to heart failure.^1^ Although the development of drug therapy and interventional technology has reduced mortality rates following MI, restoring normal cardiac structure and function remains a great challenge.^2^ In recent years, stem cell therapy, a new and potentially effective intervention, has provided new possibilities to solve this longstanding problem.

Among the various types of stem cells, mesenchymal stem cells (MSCs) have become the preferred seed cells for MI treatment because of their beneficial paracrine effects and their crucial role in both basic research and clinical trials.^3^, ^4^, ^5^ Recently, adipose tissue has emerged as a novel source of MSCs. Human adipose‐derived stem cells (hADSCs) not only possess characteristics such as rapid expansion, low immunogenicity, and multipotent differentiation potential, but they can also be easily obtained through standard liposuction procedures.^6^ Previous studies have shown that hADSCs secrete various factors that promote angiogenesis, improve immune regulation, and inhibit fibrosis.^7^, ^8^, ^9^ However, in the harsh microenvironment of ischemia, hypoxia, inflammatory reactions and local structural changes in the infarcted area, hADSCs have limitations such as limited paracrine function and a low survival rate, which significantly diminish their efficacy.^10^ Therefore, multiple studies have focused on modifying hADSCs to enhance their applications in regenerative medicine.

To enhance their therapeutic effects, hADSCs are usually genetically modified by exogenous gene transfection tailored to specific disease characteristics.^10^, ^11^ Compared with the technology based on DNA/viruses, chemically synthetic modified messenger RNA (modRNA) represents a new and efficient method of protein expression, offering advantages such as speed, safety, and low immunogenicity. This technology has already found application in clinical prophylaxis,^12^, ^13^ and its application in regenerative medicine continues to evolve. Previously, our cohort combined cell therapy with modRNA technology and achieved promising results in conditions such as lower limb ischemia,^14^ osteoarthritis,^15^ MI^16^ and corneal injury repair.^17^ As a potentially effective therapeutic strategy, the combination of modRNA and cell therapy allows for the design and integration of multiple therapeutic factors based on cell therapy, thus supporting a comprehensive and effective treatment strategy for MI.

Therapeutic factors that enhance cell therapy for MI have been extensively investigated. The combination of anti‐apoptotic and proangiogenic factors may represent a promising pharmacological approach to promoting multidimensional repair post‐MI. Preclinical studies have demonstrated the potent effects of vascular endothelial growth factor A (VEGFA) and basic fibroblast growth factor (bFGF). Several studies have shown that intramyocardial injection of modRNA encoding VEGF‐A165 induces angiogenesis.^18^, ^19^ bFGF has been shown to promote angiogenic cell proliferation in embryos and angiogenesis, both in vitro and in vivo.^20^, ^21^, ^22^ Ye et al.^23^ found that vascular smooth muscle cells promote recovery in infarcted cardiomyocytes by secreting bFGF, which activates the PI3K/Akt pathway to inhibit apoptosis and autophagy. In a separate study, Rao et al.^24^ found that administering bFGF after MI improves cardiac function and cell viability, attenuates myocardial injury and apoptosis, and enhances angiogenesis. Importantly, VEGF and bFGF are naturally expressed at low levels in hADSCs and are thought to significantly impact the regeneration and maturation of vessels and cardiac function improvement following MI.

Based on previous literature, including our own research, we proposed that hybrid therapy combining stem cells with proangiogenic and anti‐apoptotic factors could enhance the therapeutic potential of hADSCs and significantly improve cardiac function post‐transplantation. The main purpose of this study was to evaluate the therapeutic effect of hADSCs modified with VEGFA modRNA and bFGF modRNA (hADSCs^VEGFA^, hADSCs^bFGF^ and hADSCs^dual^) in promoting angiogenesis, inhibiting apoptosis, improving cardiac function, and inducing beneficial outcomes following MI. Our goal is to introduce a novel strategy for the comprehensive treatment of MI through this conceptual exploration.

## 2 MATERIALS AND METHODS

### 2.1 ModRNA synthesis and formula

ModRNA was synthesised according to previously described methods.^25^, ^26^ In short, a linear DNA template was utilised for T7 RNA polymerase‐mediated transcription. Uracil was replaced with N^1^‐methylpseudoridine in the process of in vitro transcription synthesis. RNA purification was performed using Ambion MEGAclear spin columns. Then, residual 5′‐phosphates were removed using Antarctic Phosphatase (New England Biolabs). The purity and concentration of the modRNA was evaluated by NanoDrop spectrophotometer (Thermo Fisher Scientific, Waltham, MA, USA) and suspended at a concentration of 1 µg/µL for further use.

### 2.2 Cell culture

The acquisition and use of hADSCs were approved by the Clinical Ethics Committee of Shanghai Ninth People's Hospital (No. SH9H 2018‐T22‐1). In brief, hADSCs were cultured in mesenchymal stem cell medium (ScienCell, USA) containing 5% fetal bovine serum and 1% penicillin‐streptomycin antibiotic. All cells were incubated in a humidified atmosphere of 5% CO2 at 37°C. Cells between passages 3 and 5 were used for all experiments.

### 2.3 ModRNA transfection and evaluation

ModRNA was transfected into hADSCs using Lipofectamine RNAiMAX Reagent (Invitrogen, California, USA). Liposome‐modRNA complexes were produced by mixing the properly diluted modRNA and RNAiMAX reagents. After transfecting cells for 4 h, the complexes‐containing culture medium were changed. In each well of a 6‐well plate, 4 µg of modRNA and 10 µL of RNAiMAX reagent was used to transfect approximately 1 × 10^6^ hADSCs.

Transfection efficiency was evaluated by transfecting hADSCs with green fluorescent protein (GFP) modRNA. Twenty‐four hours after transfection, the digested cells were detected using a CytoFLEX LX flow cytometer (Beckman Coulter, CA, USA).

Calcein acetoxymethyl ester (AM)/propidium iodide (PI) cytotoxicity and cell viability assay kits (Beyotime, China) were used to assess the toxicity of modRNA transfection to hADSCs. After staining with Calcein AM (1:1000) and PI (1:1000) for 15 min, hADSCs were observed using a fluorescence microscope (DMI3000 B, Leica, Heidelberg, Germany). The ImageJ 1.54f was used to perform statistical analyses.

### 2.4 Enzyme‐linked immunosorbent assays (ELISAs)

VEGFA and bFGF ELISA kits (MultiSciences, China) were employed to quantify the expression kinetics of VEGFA and bFGF of hADSCs after transfection. Conditioned media were harvested 12, 24, 48, 72, 96 120 and 168 h after transfection for analysing the protein levels of VEGFA and bFGF.

For the in vivo expression of VEGFA and bFGF, samples were harvested at 24, 72 and 168 h post cell treatments, respectively. The tissues were homogenised and centrifuged prior to content detection following the kit instructions.

Blood was drawn into clean tubes and centrifuged at 3500 rpm for 15 min. The serum was obtained and used to detect the levels of cardiac Troponin I (cTnI) and creatine kinase‐MB (CK‐MB) using ELISA kits (Elabscience, China). All tests were repeated thrice for each group.

### 2.5 Tube formation assay

To assess the effects of the conditioned medium from hADSCs transfected with luciferase modRNA, VEGFA modRNA, and/or bFGF modRNA on the tube formation ability of human umbilical vein endothelial cells (HUVECs), HUVECs were collected and inoculated into 48‐well plates coated with Matrigel (Corning, USA), then cultured in 300 µL of the abovementioned conditioned medium, respectively. Tubes were observed every 2 h. Subsequently, ImageJ was used to count the tubes and branches. All tests were repeated five times.

### 2.6 Transwell assay

To further evaluate the effects of the above‐mentioned conditioned medium from hADSCs on the migratory ability of HUVECs, HUVECs were incubated in the upper chambers of the transwell plates with an 8‐µm pore size (Corning, USA). The upper chamber of the transwell plates was inoculated with the medium of hADSCs transfected with different modRNAs, and the lower chamber was filled with medium containing 10% fetal bovine serum. After 24 h, migrated cells were fixed in 4% paraformaldehyde (PFA) and stained with 0.2% crystal violet (Aladdin, China). The migrated cells were observed using a fluorescence microscope (DMI3000 B, Leica, Heidelberg, Germany), and three randomly selected areas were used for statistical analysis.

### 2.7 Flow cytometry

H9C2 cells were seeded at a density of 1×10^5^ per well in a 12‐well plate and cultured under normoxic conditions for 24 h to allow for cell adhesion. Subsequently, the culture medium of H9C2 cells was replaced with the conditional medium. Thereafter, the cells were cultured under hypoxic conditions (1% O₂) for 30 h. FITC‐Annexin V apoptosis detection kits (Tonbo, China) were used to investigate apoptosis. The cells were analysed by flow cytometry (CytoFLEX, Beckman, Coulter). The data obtained were analysed using FlowJo 10.5 software (Tree Star Inc., Ashland, OR, USA).

### 2.8 Cell Counting Kit‐8(CCK8) assay

To evaluate the proliferation of smooth muscle cells (SMCs), SMCs were seeded into 96‐well plates at a density of 3×10^3^ cells/well and incubated in the conditioned medium of hADSCs transfected with different modRNAs. CCK8 (Dojindo, Kumamoto, Japan) assay was conducted at three time points (D0, D1, and D3). A microplate reader (Tecan i‐control, Tecan, Switzerland) was used to record the optical densities at 450 nm.

### 2.9 Animal studies

Sprague‐Dawley rats (male, 200–250 g) were provided by Shanghai Jihui Experimental Animal Co. (Shanghai, China). All animal experiments were approved by the Laboratory Animal Welfare Ethics Committee of the Shanghai Children's Medical Center (Approval No, SCMC‐LAWEC‐2019‐009). The rats were randomly divided into six groups: (1) Sham group; (2) MI group; (3) hADSCs^Luc^ group (150‐µL mixture of Matrigel (Corning) and hADSCs transfected with 20 µg Luciferase modRNA (5×10^6^ cells/rat) was injected into the left ventricular border area after MI); (4) hADSCs^VEGFA^ group (150‐µL mixture of Matrigel (Corning) and hADSCs transfected with 20 µg VEGFA modRNA (5×10^6^ cells/rat) injected into the left ventricular border area after MI); (5) hADSCs^bFGF^ group (150‐µL mixture of Matrigel (Corning) and hADSCs transfected with 20 µg bFGF modRNA (5×10^6^ cells/rat) injected into the left ventricular border area after MI); and (6) hADSCs^dual^ group (150‐µL mixture of Matrigel (Corning) and hADSCs transfected with 20 µg VEGFA and 20 µg bFGF modRNA (5×10^6^ cells/rat) injected into the left ventricular border area after MI). The rat model was established according to previously described methods.^16^ Under continuous isoflurane anaesthesia, we performed left thoracotomy and permanent ligation of the left anterior descending coronary artery with a 6‐0 thread to induce MI in the rats. During the operation, electrocardiogram (ECG) was employed to verity the successful establishment of the MI model.

For the treatment group, myocardial injections of the cells were administrated at three fixed positions at the infarct border zone 10 min following the ligation of the left anterior descending coronary artery in the rats. The modified cells were provided to the surgeon with assigned numbers only, without specific group information. The cells were injected into the recipient heart immediately after collection to maintain as much cell activity as possible. Subsequently, the chest was closed. To avoid immune rejection, all rats received 5 mg/kg/day methylprednisolone and 0.25 mg/kg/day tacrolimus every 12 h from 1 day before MI to the day they were sacrificed.

A total of 84 rats were used in the experiment. However, 6 rats died within 3 days of the model establishment. These rats showed no obvious group‐specific distribution pattern. Since no subsequent data were collected from these rats, they were excluded for further analysis.

### 2.10 Triphenyl tetrazolium chloride (TTC) staining

TTC (MP BIO, California, USA) staining was used to assess the area of the MI. Dehydrogenase present in normal myocardial tissue can reduce TTC to red water‐insoluble formazan, whereas the infarct area is white owing to a lack of dehydrogenase. Prior to staining, 2% TTC was fully dissolved in PBS. Twenty‐four hours after MI, the rats were euthanised and their hearts were collected, fully washed with precooled PBS, and then sectioned into five pieces. After stained with 2% TTC solution, the heart slices were fixed with preheated 4% PFA for 30 min. Images of the stained heart slices were taken and the area of the left ventricular infarction was analysed using ImageJ.

### 2.11 Bioluminescence imaging

Bioluminescence imaging of the transplanted hADSCs was performed using an in vivo imaging system (VISQUE® Smart‐LF, Vieworks, Korea). After the MI rat model was established, 5 ×10^6^ hADSCs transfected with VEGFA and bFGF modRNA were intramyocardially injected. After 24 h, rat hearts, livers, spleens, lungs and kidneys were harvested for imaging to evaluate the in vivo distribution of the injected cells.

### 2.12 Echocardiography

To compare the cardiac function of each group, echocardiography was performed 1, 2 and 4 weeks after MI induction. Isoflurane was used to maintain anaesthesia, and the rats were examined by a blinded technician using transthoracic echocardiography. The technician, only responsible for image acquisition and data collection, was not informed about the group assignments. A Vevo 3100 imaging system (Visualsonics, Toronto, Canada) and a MS‐250 transducer were used to record left ventricular systolic and diastolic movements. The left ventricular end‐systolic volume (LVESV) and left ventricular end‐diastolic volume (LVEDV) were automatically calculated. Cardiac function was assessed by left ventricular ejection fraction (LVEF) and left ventricular short‐axis shortening (LVFS), which were calculated using the following equations:

$$\begin{array}{ccc} \mathrm{LVEF}\;(\%) & = & ((\text{LVEDV-LVESV})/\text{LVEDV})\times 100\%,\\ \mathrm{LVFS}\;(\%) & = & ((\text{LVIDd-LVIDs})/\text{LVIDd})\times 100\%. \end{array}$$

### 2.13 Epicardial activation mapping

The rats were anaesthetised 15 min and 4 weeks following MI surgery. Tracheal intubation and isoflurane were used to maintain anaesthesia. After left thoracotomy, 8 × 8 microelectrodes (MappingLab Inc., UK) were placed on the epicardial surface to record the epicardial electrical activity. The obtained active waveform was amplified using a filter amplifier (MappingLab Inc., UK) and then transmitted to a computer. All activation times were digitised and used to draw activation diagrams. Activation time was calculated as the maximum negative slope point of the active waveform. The conduction time (CT), conduction velocity (CV) and non‐uniformity index were analysed using EMapScope 4.0 software (MappingLab Inc., UK).

### 2.14 Histology and immunohistochemical staining

Rat hearts were collected 1 and 4 weeks after MI. Haematoxylin‐eosin staining (Solarbio, China) and Masson's trichrome staining (Solarbio, China) were performed. Scar tissue (%) and LV wall thickness of the heart were calculated using ImageJ.

A one‐step terminal deoxynucleotidyl transferase dUTP nick end labelling (TUNEL) apoptosis detection kit (Servicebio) was used for TUNEL staining following the manufacturer's protocol. In short, the slices were hydrated with xylene and a series of graded ethanol solutions and stained with TUNEL reagent. Finally, the slices were counterstained with DAPI. Images were obtained using a laser scanning confocal microscope (TSC SP8, Leica).

For immunohistochemistry, antigen retrieval was performed using citric acid buffer (Yeasen, China). After being permeabilised with 0.2% Triton X‐100 (Beyotime, China) and blocked with 5% bovine serum albumin, the heart slices were incubated with the primary antibodies at 4°C overnight and then stained with the secondary antibody at RT for 2 h. Finally, the samples were stained with DAPI (Yeasen, China). The primary antibodies used in this study included CD31 (ab182981, Abcam), Ki‐67 (ab16667, Abcam), cTnT (ab8295, Abcam) and α‐SMA (ab7817, Abcam). A confocal laser‐scanning microscope (Leica; TSC SP8) was used to record the results.

### 2.15 Statistical analysis

All data are shown as mean ± standard deviation (SD). Prior to further statistical analyses, the normality of the data distribution was examined using Shapiro–Wilk test. Depending on the data type, Student's *t*‐tests, one‐way analyses of variance (ANOVAs) followed by Tukey's test, or two‐way ANOVAs followed by the Bonferroni post hoc test (GraphPad Software, San Diego, CA, USA) were performed. The results were obtained from at least three independent experiments, and a value of *p* < .05 was deemed as statistically significant.

## 3 EXPERIMENTAL RESULTS

### 3.1 Efficient transfection of modRNA in hADSCs

**FIGURE 1:**
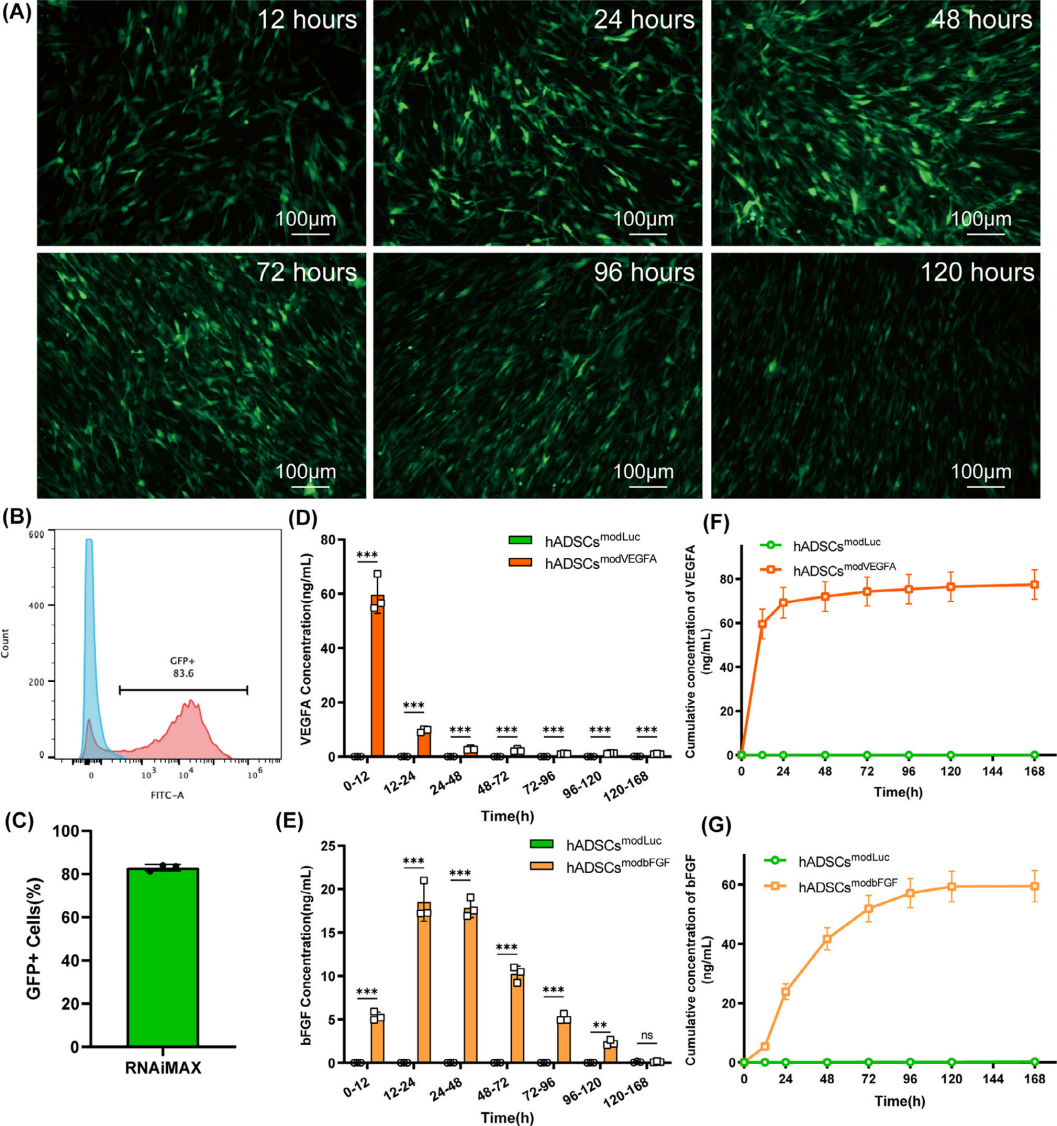
Efficiency of modRNA transfection in hADSCs. (A) Fluorescence microscopy shows that the signal of hADSCs transfected with modGFP existed from 12 to 120 h after transfection, scale bar = 100 µm. (B, C) 24 h after transfection, transfection efficiency was detected by flow cytometry. (D) Kinetics of the newly secreted VEGFA protein in the culture medium of hADSCs following VEGFA modRNA transfection. (E) Kinetics of the newly secreted bFGF protein in the culture medium of hADSCs following bFGF modRNA transfection. (F) Cumulative amount of VEGFA protein secreted by hADSCs after VEGFA modRNA transfection.(G) Cumulative amount of bFGF protein secreted by hADSCs after bFGF modRNA transfection. *p < .05, **p < .01, ***p < .001.

The GFP reporter gene was used to verify the expression and transfection efficiency of modRNA in hADSCs. After 24 h, GFP protein expression level was detected using flow cytometry. The expression of GFP protein lasted for at least 5 days following transfection (Figure 1A). Furthermore, the transfection efficiency of modRNA reached 82.93% ± 1.5% within the first 24 h after transfection (Figure 1B and C). In addition, AM/PI staining showed that transfection with the modRNA had no significant effect on cell viability (Figure S1A and B).

To detect whether hADSCs were effectively transfected with VEGFA and bFGF modRNAs and determine their expression dynamics, we transfected luciferase, VEGFA and bFGF modRNAs and monitored protein expression levels in hADSCs. Since VEGFA and bFGF are secreted proteins, we used ELISAs to measure the levels of newly generated and accumulated proteins in the cell culture medium of hADSCs transfected with VEGFA and bFGF modRNAs. We found that hADSCs transfected with VEGFA or bFGF modRNA began to rapidly secrete VEGFA or bFGF protein, respectively, for the first 2 days and continued to express them for more than 5 days. However, by the seventh day, the expression of VEGFA and bFGF proteins in the transfected hADSCs diminished substantially (Figure 1D–G). Secretion in the transfection group was significantly higher than that of the control group at all time points (Figure 1D and E). These results indicate that modRNAs can efficiently transfect hADSCs and express the target protein rapidly and efficiently following transfection.

### 3.2 In vitro function of proteins secreted by modRNA‐modified hADSCs

**FIGURE 2:**
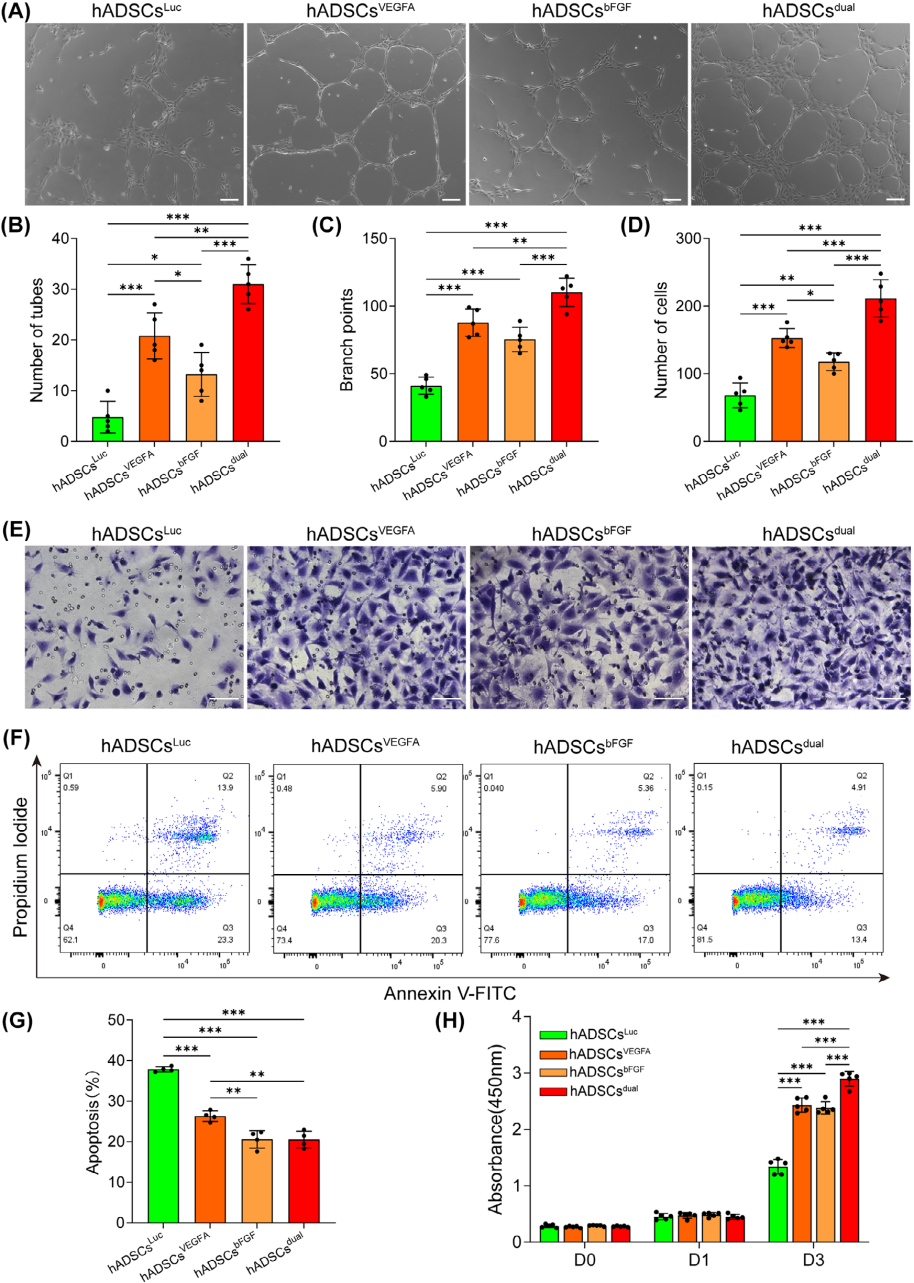
In vitro biological function of modRNA‐transfected hADSCs. (A) Supernatants from various modRNA transfection groups promoted the formation of small tubular structure of HUVECs, scale bar = 100 µm. (B) Analysis of the number of formed tubes. (C) Quantification of branch points in the tubular structures. (D) Analysis of the migrated cells. (E) Supernatants from different modRNA transfection groups promoted the migration of HUVECs, scale bar = 100 µm. (F) Flow cytometric analysis of apoptosis of H9C2 cultured in the supernatant from different modRNA transfection groups using annexin V/PI. (G) Quantification of the ratio of apoptotic cells. (H) Supernatant from different modRNA transfection groups promoted the proliferation of SMCs. *p < .05, **p < .01, ***p < .001.

To evaluate the effects of VEGFA and bFGF modRNA transfection on HUVECs, we quantified the tube formation and migration abilities of HUVECs cultured under different conditions. Compared with the hADSCs^Luc^ group, the hADSCs^VEGFA^, hADSCs^bFGF^ and hADSCs^dual^ groups promoted the formation of the tubular structure of HUVECs (Figure 2A–C). The ability of tube formation of the conditioned medium in the hADSCs^dual^ group was significantly higher than that in the other groups (Figure 2B and C). Consistent with the results of the tube‐formation experiment, the transwell assay also showed that the conditioned medium of the hADSCs^dual^ group increased the migration ability of HUVECs more strongly than that of the other groups (Figure 2D and E). The above results indicated that hADSCs transfected with VEGFA and bFGF modRNAs, both showed strong angiogenic characteristics in vitro, and the combination of the two modRNAs was more efficacious, which further confirmed their therapeutic potential in heart therapy.

Next, conditioned media from different modRNA transfection groups were used to detect their effect on H9C2 apoptosis under hypoxia. We found that, compared to the hADSCs^Luc^ group, transfection with VEGFA or bFGF modRNA significantly reduced the percentage of apoptotic H9C2 cells under hypoxia. Compared to the hADSCs^VEGFA^ group, the hADSCs^bFGF^ and hADSCs^dual^ groups significantly inhibited H9C2 apoptosis (Figure 2F and G). These results suggest that the bFGF modRNA exerts a stronger inhibitory effect on H9C2 apoptosis than VEGFA modRNA.

To evaluate the effect of the conditioned medium of hADSCs transfected with VEGFA and bFGF modRNA on SMCs proliferation, cell counting was performed at 0, 1 and 3 days after SMC inoculation to quantify cell proliferation. The results showed that on the third day, the hADSCs^VEGFA^, hADSCs^bFGF^ and hADSCs^dual^ groups significantly promoted the proliferation of SMCs and the hADSCs^dual^ group showed the most favourable results (Figure 2H).

### 3.3 hADSCs^dual^ improved cardiac function following MI in vivo

**FIGURE 3:**
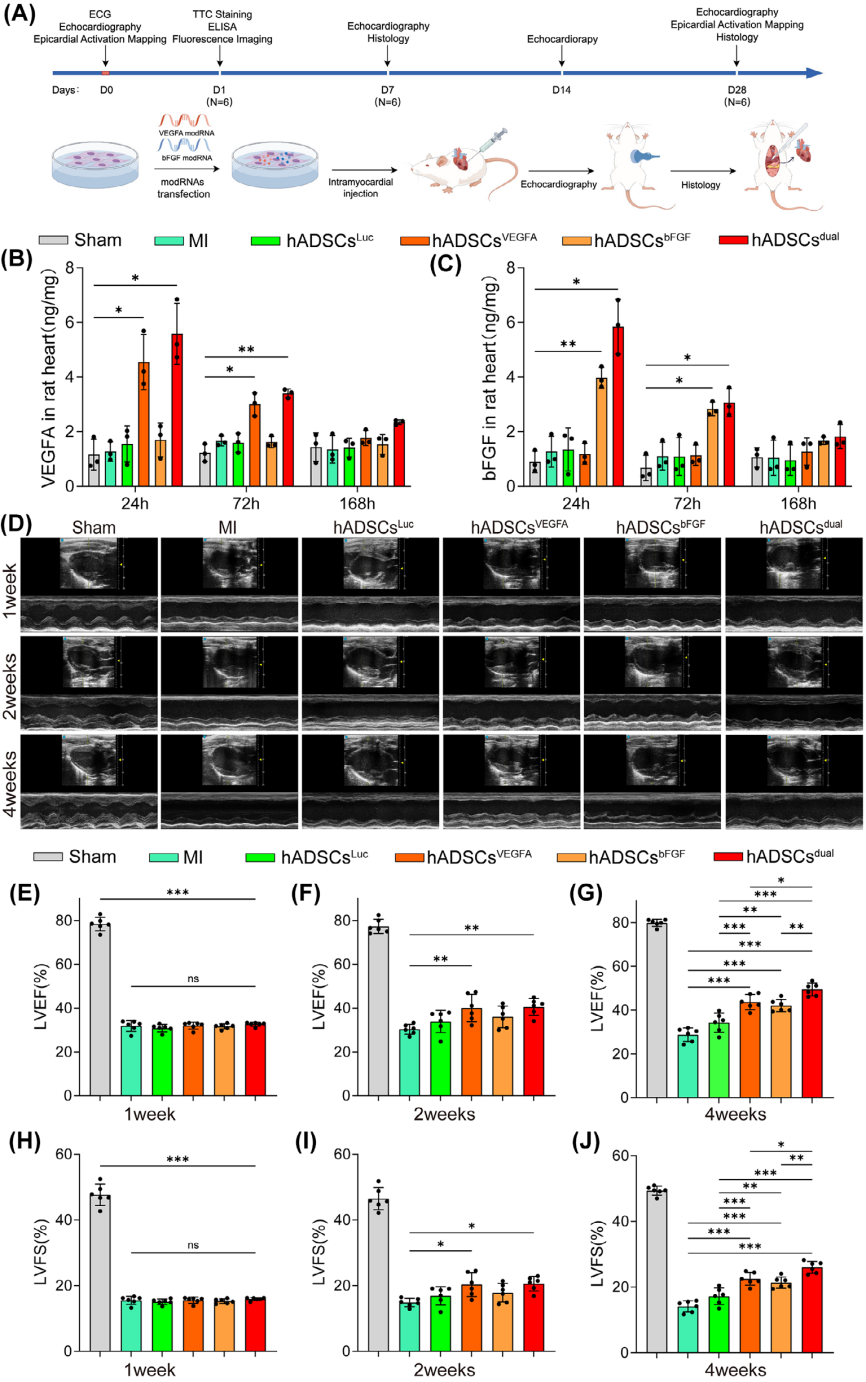
Recovery of cardiac function following hADSCs transplantation. (A) Schematic diagram illustrating the surgical protocol, in vivo research design, and time points for experimental data collection. Figure created by FigDraw. (B, C) Expression of VEGFA and bFGF modRNA in the rat hearts after hADSCs transplantation. (D) M‐mode echocardiography of rats at different time points (1, 2 and 4 weeks after MI induction, respectively). (E–G) Analysis of LVEF post‐MI. (H–J) Analysis of LVFS after MI induction. ns indicates *p* > .05, *p < .05, **p < .01, ***p < .001.

Next, we investigated the potential of modRNA‐transfected hADSCs for MI treatment. Animal experimental procedures are shown in Figure 3A. All rats that underwent surgery experienced transmural MI. Typical changes were expressed in the ECG of rats at 5 min after ligation of the left anterior descending coronary artery (LAD). In comparison to the ECG prior to ligation, ECG of rats after LAD ligation exhibited an elevation of the ST segment (Figure S2A). Cardiac ultrasound detection showed that the ejection fraction in the MI group was significantly decreased to a range between 25% to 35% (Figure S2B). Compared with the sham group, 24 h after the model was established, CK‐MB and cTnI serum levels in the MI group were remarkably elevated, with an increase of more than tenfold (Figure S2C and D). TTC staining showed that the colour boundary between the infarcted and normal myocardium was clear, and the percentages of the infarct areas of the left ventricle were stable at 37% (Figure S2E and F).

To evaluate the safety of hADSCs, a series of in vivo experiments were conducted. The in vivo distribution of hADSCs was examined 24 h after the transplantation procedure. The results demonstrated that at 24 h after the surgical procedure, the hADSCs were principally distributed within the heart, while no prominent presence of hADSCs was discernible in the liver, spleen, lungs or kidneys (Figure S3A). HE staining was also performed. The histological examination under microscopy did not show any overt pathological alterations (Figure S3B), which implied the satisfactory biocompatibility and safety of hADSCs transplantation, thus providing a basis for further investigation into their long‐term behaviours and therapeutic efficacies.

The expression efficacies of VEGFA and bFGF modRNA in the infarcted zone are a prerequisite for their therapeutic efficacy. ELISA kits were employed to detect the expression levels of VEGFA and bFGF proteins in rat hearts. We measured the protein contents of VEGFA and bFGF at 24, 72 and 168 h postoperatively. The results indicated that during the first 72 h after transplantation, the expression of the target proteins in the hADSCs^dual^ group was significantly higher than that in the MI group (Figure 3B and C). However, at 168 h, there was no significant difference in the protein levels between the hADSCs^dual^ group and the MI group (Figure 3B and C).

To confirm whether modRNA‐modified hADSCs affected LV function, we performed a series of echocardiographic measurements after MI surgery and cell treatment. Specifically, the following six groups were established: sham, MI, hADSCs^Luc^, hADSCs^VEGFA^, hADSCs^bFGF^ and hADSCs^dual^ groups. Echocardiography was performed on rats from different groups 1, 2 and 4 weeks after MI and treatment (Figure 3D). Echocardiography revealed that the LV function of rats in each group decreased significantly 1 week after the MI operation, and the extent of decline was comparable among all groups at this time point (Figure 3E). Cardiac ultrasound detection showed that there was no statistically significant difference in the LVEF and LVFS in each group in the first week after MI with/without cell transplantation (Figure 3E and H). However, 2 weeks after MI induction, LVEF and LVFS in rats treated with hADSCs^VEGFA^ and hADSCs^dual^ increased significantly in the hADSCs^VEGFA^ and hADSCs^dual^ groups compared with other MI groups (Figure 3F and I). At the fourth week, the LV function of rats in the hADSCs^dual^ group was significantly improved, LVEF increased from 32.71% ± .93% to 49.43% ± 2.90%, and LVFS increased from 15.94% ± .45% to 26.05% ± 1.79% (Figure 3G and J). The LV function in rats that received hADSCs^dual^ was found to be significantly higher than that of rats that received either hADSCs^VEGFA^ or hADSCs^bFGF^ treatments. The LV function of hADSCs^VEGFA^ improved slightly to 43.72% ± 3.46%, while LVFS increased to 22.51% ± 1.96% (Figure 3G and J). The LV function of the hADSCs^bFGF^ group slightly improved to 42.10% ± 2.80%, while LVFS increased to 21.38% ± 1.71% (Figure 3G and J). The treatment effects of modRNA‐modified hADSCs all exceeded that of the non‐cell‐based treatments. Echocardiographic parameters increased significantly in rats receiving hADSCs^dual^, illustrating the therapeutic potential of hADSCs^dual^ as a novel cardiovascular cell therapy for improving cardiac dysfunction after MI.

### 3.4 hADSCs^dual^ promoted left ventricular electrical conduction after MI in vivo

**FIGURE 4:**
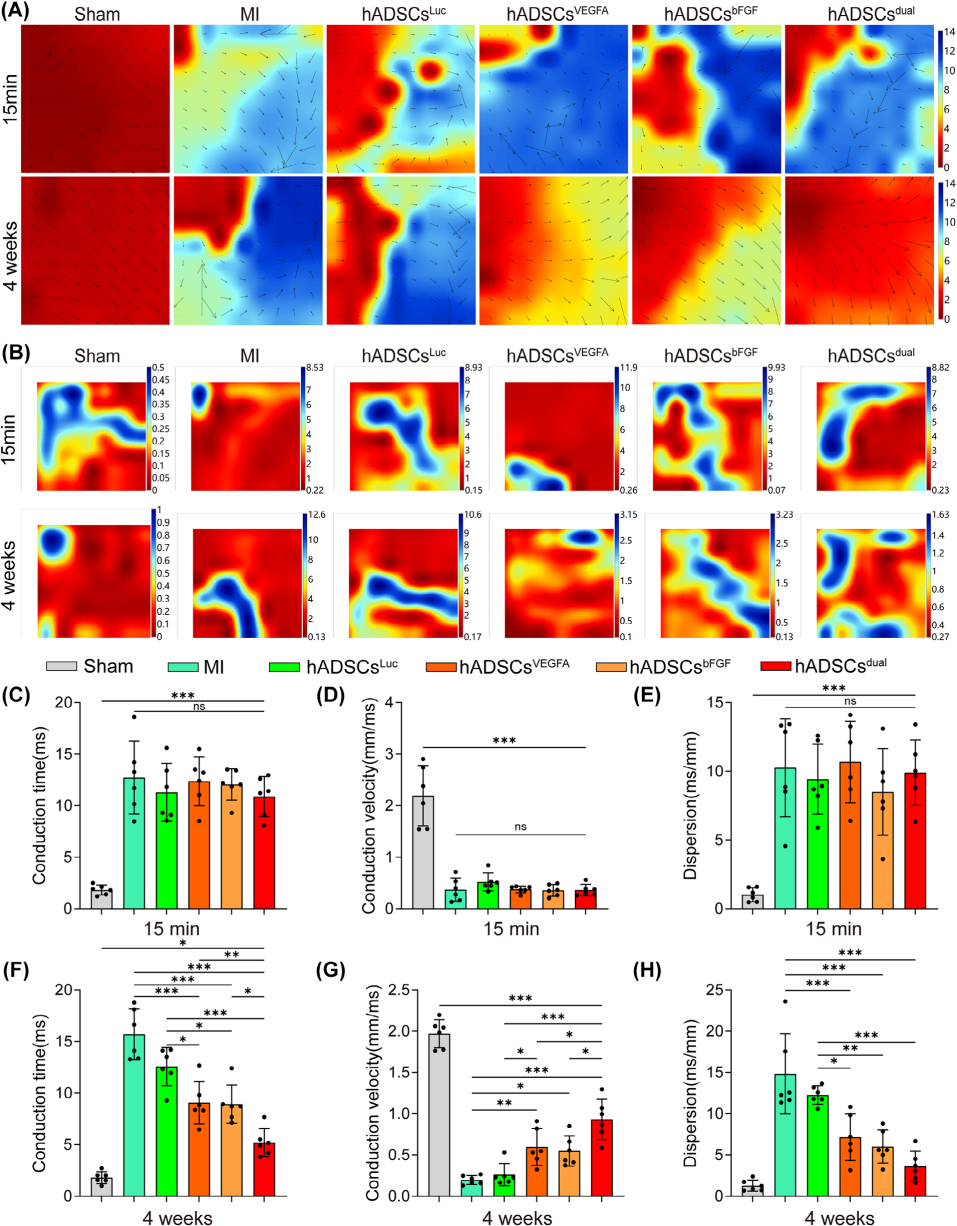
Recovery of left ventricular electrical conduction function following hADSCs transplantation. (A) Representative electrophysiological images of left ventricular spontaneous conduction at 15 min and 4 weeks after MI with/without treatment (from red to blue: bar graph represents the total time of a heartbeat from the first to the last measurement). (B) Representative images of left ventricular conduction dispersion at 15 min and 4 weeks after MI surgery with/without treatment. (C–E) Quantification of conduction time (C), conduction velocity (D), and conduction dispersion (E) at 15 min post‐MI modelling. (F–H) Quantification of conduction time (F), conduction velocity (G), and conduction dispersion (H) at 4 weeks after MI surgery with/without treatment. ns indicates *p* > .05, *p < .05, **p < .01, ***p < .001.

To evaluate changes in left ventricular electrical conductivity after MI surgery and treatment interventions, we measured the left ventricular electrical conduction at 15 min and 4 weeks post‐MI. Compared to the sham group, all groups showed disordered conduction (Figure 4A), and the dispersion of electrical activity significantly increased at 15 min post‐MI surgery (Figure 4B). At the fourth week, compared to the MI group and hADSCs^Luc^ group, rats receiving hADSCs^VEGFA^, hADSCs^bFGF^, or hADSCs^dual^ showed more orderly conductivity (Figure 4A), and the dispersion of electrical activity decreased (Figure 4B). Quantitative analysis of CT, CV, and dispersion of electrical activity in different groups was performed 15 min and 4 weeks after MI surgery and intervention treatment. We found that after MI surgery, the CT was prolonged, CV was significantly reduced, and conduction dispersion increased (Figure 4C–E). After treatment with hADSCs^VEGFA^, hADSCs^bFGF^ or hADSCs^dual^ for 4 weeks, the CT of the left ventricle became shorter, the CV increased, and the dispersion of conduction decreased (Figure 4F–H). Compared to the other groups, the left ventricular conduction parameters of the hADSCs^dual^ group were significantly improved, supporting the therapeutic potential of hADSCs^dual^ as a new treatment method to improve left ventricular conductivity.

### 3.5 hADSCs^dual^ reduced cardiac fibrosis and preserved ventricular wall thickness

**FIGURE 5:**
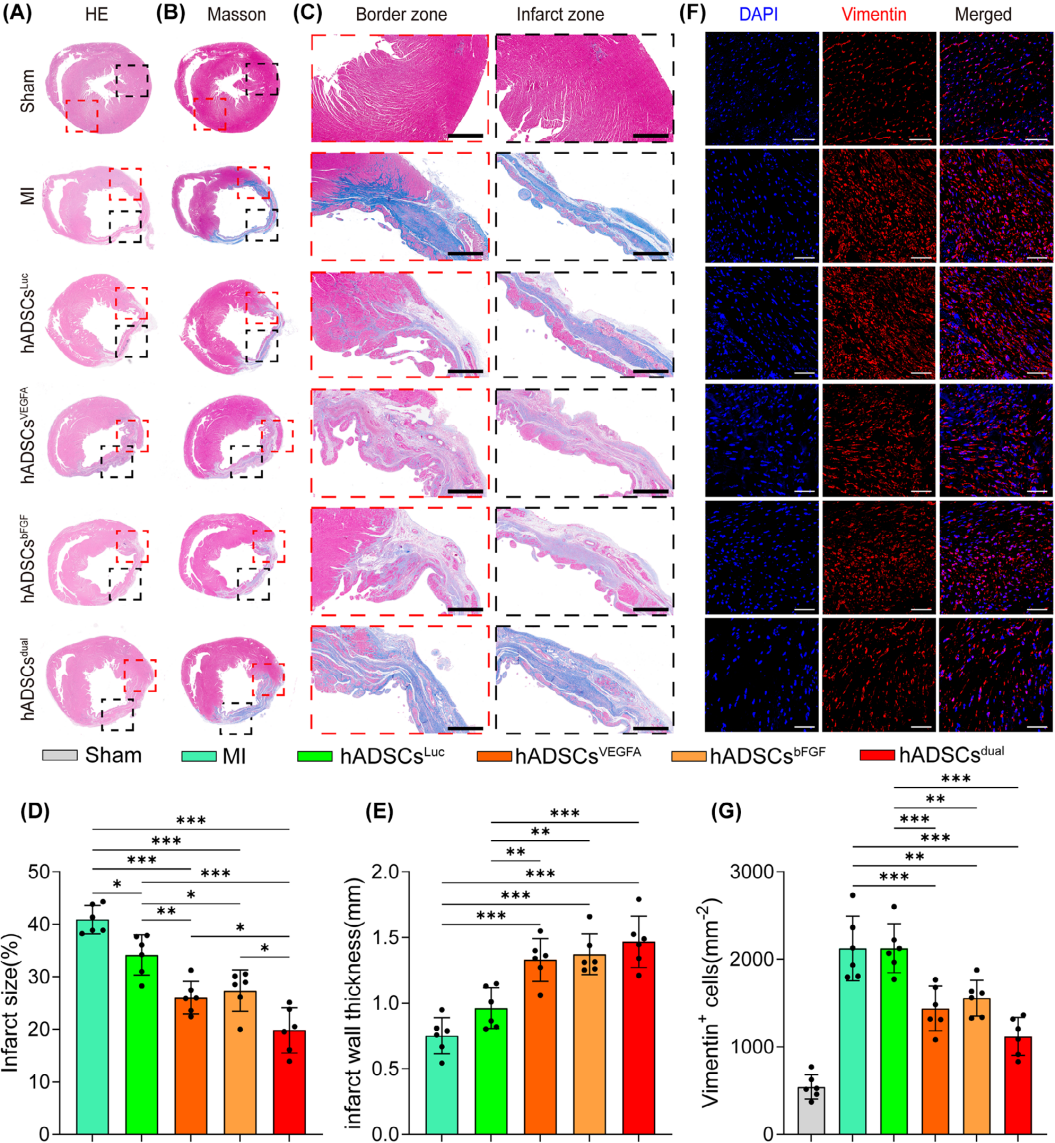
Ventricular morphological analysis of rats following MI and treatment. (A, B) Representative images of haematoxylin and eosin or Masson's trichrome‐stained cross‐sections of the infarcted ventricular myocardium at 4 weeks after MI surgery with/without treatment. In (B), the red box indicates the border zone, and the black box indicates the infarct zone. (C) Magnified images of the border zone and infarct zone stained by Masson's trichrome in each group, scale bar = 50 µm. (D) Analysis of infarct size in different groups. (E) Comparisons of left ventricular wall thickness in each group. (F) Representative images of left ventricular Vimentin immunostaining of infarcted rats in each group, scale bar = 50 µm. (G) Analysis of vimentin^+^ cells. * *p* < .05, **p < .01, ***p < .001.

The hearts were harvested 4 weeks after modelling. Haematoxylin‐eosin staining confirmed that, compared with other groups, the proportion of regenerated tissue in the heart of rats treated with hADSCs^dual^ was significantly increased (Figure 5A). The left ventricular scar area of rats, as detected by Masson staining, was significantly smaller in the hADSCs^dual^ group, and the ventricular wall thickness was well‐maintained (Figure 5B and C). Quantitative analysis of the left ventricular infarction area and wall thickness of rats in each group also confirmed this finding (Figure 5D and E). Vimentin staining also revealed numerous fibroblasts in the infarcted zone in all groups 4 weeks post‐MI (Figure 5F). However, compared to the MI group, the hADSCs^dual^ group showed fewer infiltrating fibroblasts (Figure 5F and G). Overall, hADSCs^dual^ reduced cardiac fibrosis, alleviated ventricular remodelling and inhibited the progression of myocardial injury induced by MI to a certain extent.

### 3.6 hADSCs^dual^ reduced apoptosis and promoted cell proliferation in the infarcted area

Previous studies have shown that MSCs exert their therapeutic effects primarily through paracrine signalling; therefore, we investigated the survival of hADSCs after implantation in vivo. To compare the engraftment between hADSCs^dual^ and hADSCs^Luc^, we used CMDIL^+^ hADSCs to exert their therapeutic effects. hADSCs were found in all rats 1 and 4 weeks following MI surgery and hADSCs treatment (Figure S4A and B). Additionally, most hADSCs gathered in the infarct and border zones, suggesting minimal migration following injection. Interestingly, a significantly larger percentage of hADSCs pretreated with VEGFA and/or bFGF modRNA survived in the infarcted zone (Figure S4C and D).

**FIGURE 6:**
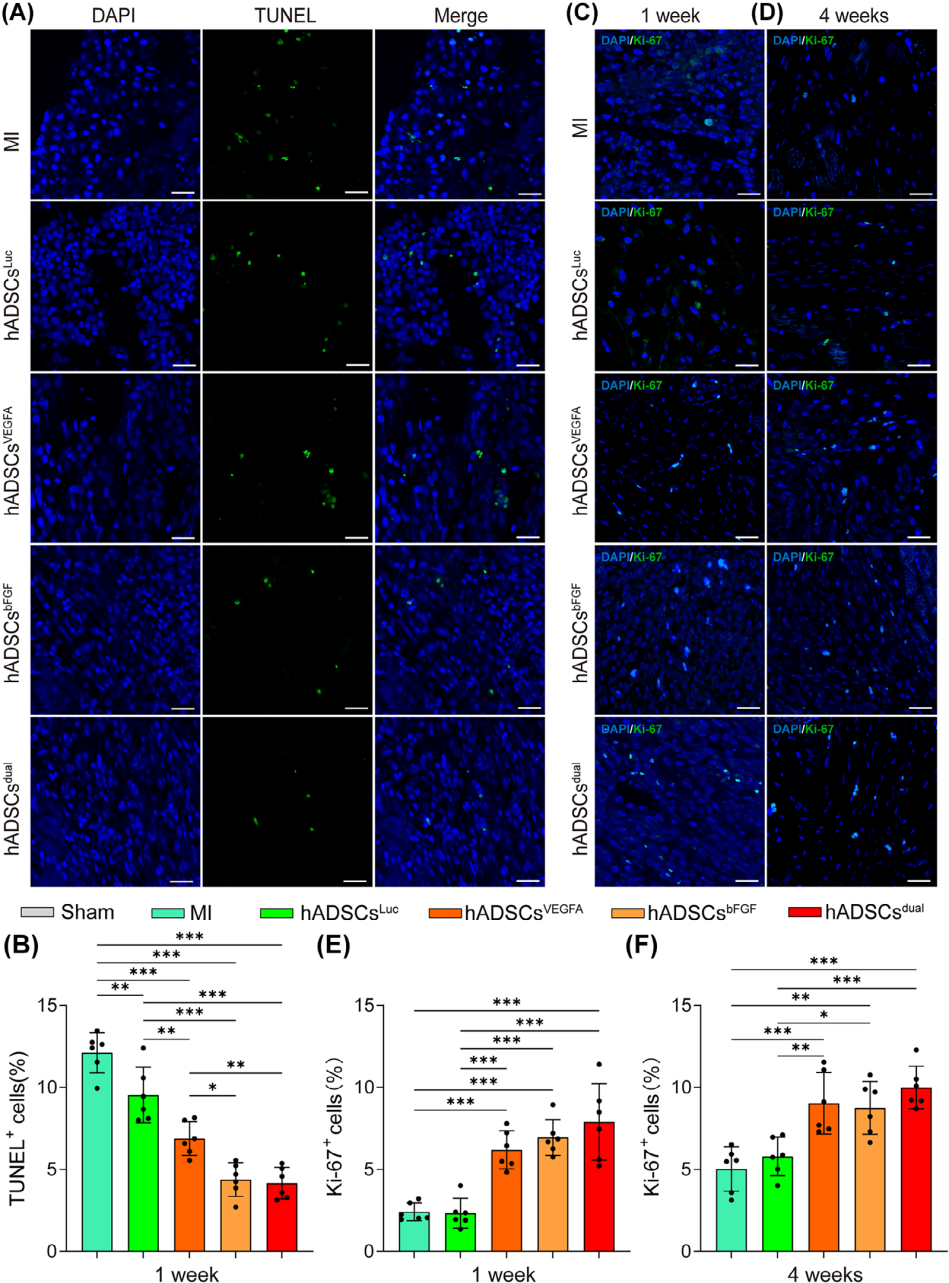
Apoptosis and proliferation in the infarction zone after MI and hADSCs transplantation. (A) Representative images of TUNEL‐stained cross‐sections of the infarcted ventricular myocardium at 1 week after MI surgery with/without treatment, scale bar = 25 µm. (B) Comparisons of apoptosis in infarcted area of rats among each group. (C, D) Representative images of Ki‐67‐stained cross‐sections of the infarcted ventricular myocardium at different time points after MI surgery with/without treatment, scale bar = 25 µm. (E, F) Comparisons of Ki‐67^+^ cells of rats at different time points. *p < .05, **p < .01, ***p < .001.

To gain further insights into the mechanism of cardiac function improvement and fibrosis reduction, we first assessed the apoptosis of cells in the infarct region. Treatment with hADSCs^dual^ inhibited apoptosis in the infarcted zone. As shown by the TUNEL staining results, compared with the MI group, the proportion of apoptotic cells in the infarct zone in the hADSCs implantation group decreased to a certain extent 1 week following treatment (Figure 6A). Compared with the hADSCs^Luc^ group (9.54 ± 1.70%), the hADSCs^VEGFA^ group (6.88 ± 1.03%), hADSCs^bFGF^ group (4.39 ± 1.03%), and hADSCs^dual^ group (4.17 ± .95%) showed significantly fewer apoptotic cells in the infarcted area (Figure 6B). In addition, Ki‐67 staining was performed to assess cell proliferation in the infarcted zone 1 and 4 weeks following MI surgery and treatment (Figure 6C and D). Compared to the hADSCs^Luc^ group, hADSCs^dual^ significantly promoted cell proliferation in the infarcted zone (Figure 6E and F). To further clarify the types of cells undergoing apoptosis and proliferation, immunostaining for cardiac Troponin T (cTnT) was carried out. Although we detected TUNEL‐positive cells in the vicinity of residual cardiomyocytes expressing cTnT, along with a small number of cells showing co‐expression of cTnT and Ki‐67 (Figure S5A and B), the specific cell types were difficult to identify.

### 3.7 hADSCs^dual^ promoted stable vascular regeneration in vivo

**FIGURE 7:**
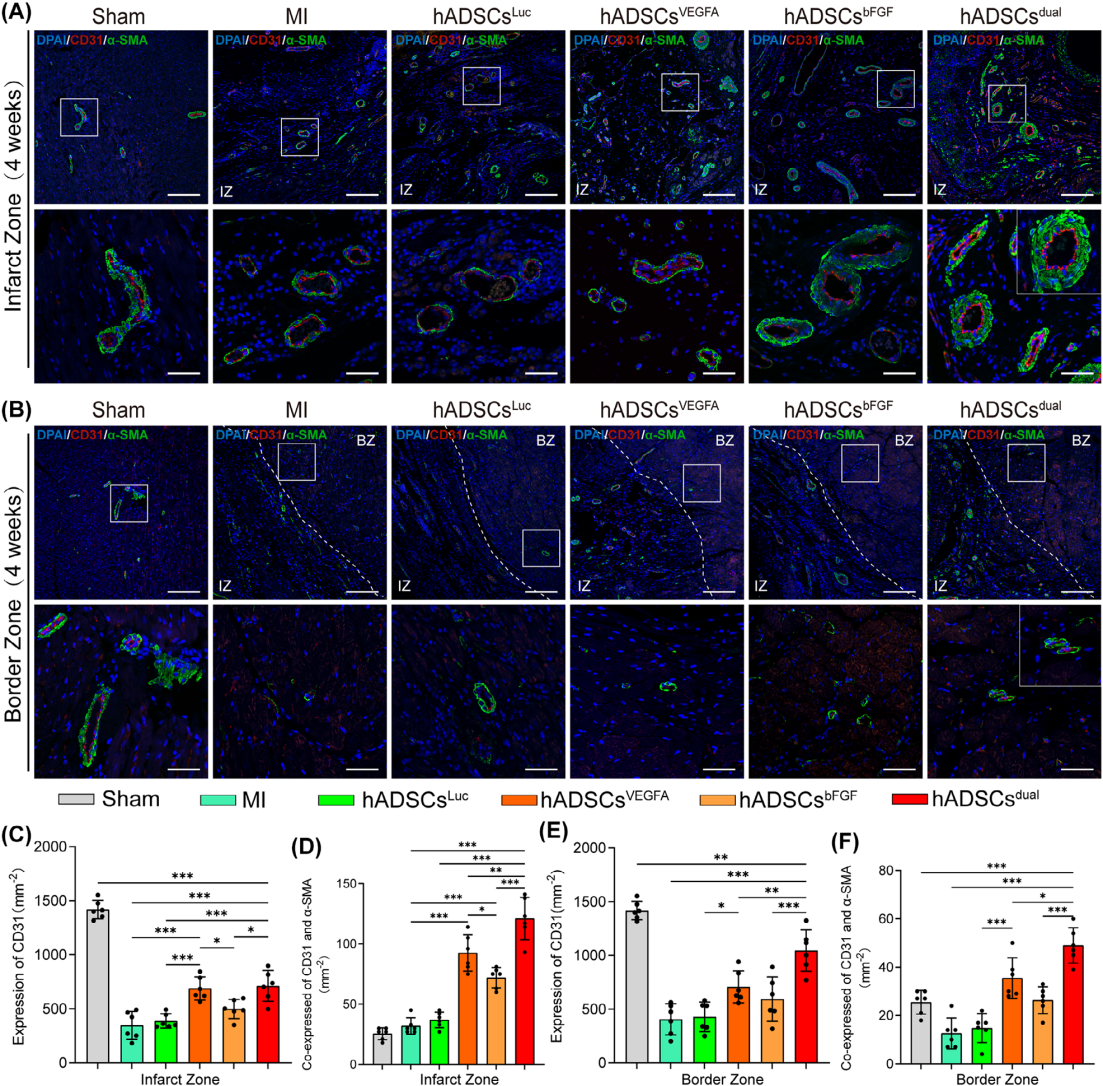
Neovascularisation of the infarct zone and border zone following 4 weeks post‐transplantation. (A, B) Confocal microscopic images showed the expressions of α‐SMA and CD31 in the infarct zone (IZ) and the border zone (BZ). Scale bar = 200 µm; zoomed‐in scale bar = 50 µm. (C–F) Analysis of the density of the capillary expressing CD31 only and the density of mature vessels expressing CD31 and α‐SMA in the infarct area (C, D) and border area (E, F). *p < .05, **p < .01, ***p < .001.

To verify the capacity of hADSCs^dual^ to promote neovascularisation within the grafted region and whether hADSCs^dual^ can better promote angiogenesis in the infarct region, immunofluorescence double‐staining with CD31 and α‐SMA staining were conducted. The staining results showed that, compared with the MI and luciferase modRNA transfection groups, the number of blood vessels in the infarcted area was significantly greater after treatment (Figure 7A). hADSCs^VEGFA^ was superior to hADSCs^bFGF^ in promoting vascular regeneration, indicating that VEGFA played a more critical role than bFGF in promoting vascular regeneration. Interestingly, we observed that most of the newly formed vessels in the MI area co‐expressed CD31 and α‐SMA in the rats treated with hADSCs^dual^, indicating mature blood vessels formation (Figure 7A, C and D). In addition, we found that the density of capillaries and mature vessels in rats receiving hADSCs^dual^ were significantly increased at the border zone of the MI area, when compared with those of all other treatments (Figure 7B, E and F).

## 4 DISCUSSION

In this study, we used two types of modRNAs to enhance cell therapy for acute MI in rats. Our results showed that hADSCs^dual^ effectively promoted the recovery of cardiac function and left ventricular conduction. Histological results demonstrated a reduction in fibrotic area, increased density of newly formed blood vessels, and alleviation of ventricular remodelling. Our study emphasises that combining hADSCs with modRNAs promotes comprehensive repair following MI.

Several studies have reported that both autologous and allogeneic mesenchymal stem cell transplantation can improve cardiac function in different MI animal models.^27^, ^28^ An injection of ADSCs can repair damaged myocardium post‐MI, improve left ventricular function and remodelling, and enhances neovascularisation through various paracrine cytokines. This approach is considered a potential treatment method.^29^, ^30^ However, due to the complex systemic microenvironment following MI—characterised by inflammation, immune responses, fibrosis, ischemia, and other pathological changes—the effectiveness of existing cell therapies remains limited. To broaden their applicability, cells need to undergo modification through various methods to enhance their therapeutic effects.^31^ Considering that the therapeutic effect of hADSCs is largely attributed to their secretion of paracrine factors, it is important to explore ways to accelerate their action and promote efficacy in preventing the progression of acute MI injury.^32^, ^33^

To improve the therapeutic potential of hADSCs and the prognosis of MI, modRNA technology was used to enhance hADSCs with the VEGFA/bFGF modRNA. Previous studies have shown that intra‐infarct administration of VEGFA significantly promotes vascular density in the infarcted area, reduces tissue necrosis and improves cardiac function.^16^, ^34^, ^35^ In addition, several clinical studies have also confirmed the beneficial effects of the local application of VEGFA, which has been shown to improve myocardial perfusion and function with sustained symptomatic relief in end‐stage angina pectoris.^36^, ^37^ These studies indicate that VEGFA plays a pivotal role in promoting angiogenesis. However, VEGFA primarily acts at an early stage of angiogenesis, leading to the formation of immature blood vessels that exhibit increased permeability, and compromising functionality after application alone.^38^, ^39^ To promote the maturation and stabilisation of neovascularisation, employing a combination of multiple factors may be a feasible approach.^34^, ^38^ bFGF is involved in the regulation of cell survival, division, differentiation, and migration, contributing to angiogenesis.^40^, ^41^ A previous study reported that bFGF application significantly increases vascular density, decreases myofibroblast density, enhances cardiomyocyte survival, and improves cardiac function.^42^ Furthermore, low‐dose bFGF and VEGFA can improve collateral circulation in dogs with acute hind limb ischemia.^43^ Though VEGFA and bFGF plasmids, or recombinant proteins, have shown promising results in improving vascular regeneration in preclinical studies, their effectiveness in clinical trials has often been limited,^44^, ^45^, ^46^ emphasising the need for novel drug delivery platforms.

In our study, we combined modRNA technology with cell therapy and used two factors to enhance the function of hADSCs to achieve a better recovery effect. This combination shows promising applications in therapy. ModRNA technology enhances the expression of specific cytokines and growth factors within cells and exerts strong therapeutic effects. Different combinations of modRNAs provide flexible options for cell therapy. Furthermore, cells enable rapid and efficient expression of modRNAs by providing essential chemicals and energy during protein synthesis.^14^, ^47^, ^48^ In this study, we verified successful modRNA expression in hADSCs, demonstrating the tolerance to modRNA overexpression (Figure 1). This observation was further confirmed by evaluating the cellular activity of hADSCs‐overexpressing modRNA (Figure S1). In addition, transient overexpression of the VEGFA/bFGF protein promoted the tubular formation and migration characteristics of HUVECs while inhibiting apoptosis in H9C2 cells (Figure 2). These results demonstrate the advantages of combined modRNA therapy.

To further explore the therapeutic potential of our combined treatment strategy, we established a rat model of acute MI and systematically evaluated the therapeutic effects of hADSCs^dual^. Our results demonstrate that hADSCs^dual^ improved cardiac function, reduced infarct size and fibrosis, and alleviated ventricular remodelling post‐MI (Figure 3). In addition, myocardial electrical conduction is crucial for repairing damaged myocardium. We analysed the direction, CT and dispersion of ventricular electrical conduction in infarcted myocardium both at 15 min and 4 weeks post‐MI. The results showed that hADSCs^dual^ promoted orderly cardiac electrical conduction and improved electrical stability post‐MI (Figure 4). These results indicate that hADSCs^dual^ is a promising new strategy that could potentially provide an effective method for the comprehensive treatment of MI.

To gain more extensive insights into the mechanisms underlying the repair of injured myocardium after acute MI in response to hADSCs^dual^ use, we first assessed cell survival and angiogenesis in the infarcted area. Previous research indicates that the efficacy of hADSCs primarily relies on their paracrine effects^49^, ^50^, ^51^ rather than their ability to differentiate into myocardial tissue and couple with host cardiomyocytes in vivo.^52^ Thus, ensuring cell survival is key to their therapeutic efficacy. In this study, compared with the hADSCs^Luc^ group, a greater number of hADSCs were observed in the experimental groups, indicating a better paracrine effect. Most hADSCs gathered in the infarct and border zones and did not integrate into the host myocardial cells (Figure S4). In addition, previous studies have shown that VEGFA alone can lead to the formation of immature vessels with increased permeability, whereas the combined application of growth factors may promote their vascular stability.^38^, ^39^ Asahara et al.^53^ found that compared with either factor alone, the combined administration of VEGF and bFGF stimulated greater collateral circulation, resulting in superior haemodynamics. This is vital for the treatment of severe arterial insufficiency not amenable to direct revascularisation. Tomanek et al.^54^ demonstrated that VEGFA and bFGF contributed to establishing a normal hierarchical structure in small arteries during the early neonatal period. Though both VEGFA and bFGF promote capillary formation, bFGF plays a greater role in the formation of small arteries. Another study has confirmed the synergistic effects of VEGF and bFGF.^55^ In this study, we observed a similar phenomenon. The treatment of hADSCs with VEGFA and bFGF mRNA significantly increased the amount of CD31^+^/α‐SMA^+^ co‐expressing vessels (Figure 7). These results led us to hypothesis that the extensive neovascular networks observed within the infarct and border zones following hADSCs^dual^ treatment were likely to prevent progressive remodelling and relieve myocardial dysfunction.

Our study has a few limitations that should be acknowledged. First, our animal experiments used male rats, which may have introduced a sex‐related bias, potentially limiting the generalisability of our results. In addition, further comprehensive control experiments are required to determine the optimal dose and ratio of modRNA and to establish the advantages of our treatment platform over other modRNA delivery methods. To improve their applicability, future studies should explore the efficacy of cell‐free products such as exosomes as therapeutic carriers or explore additional types of factors in combination with comprehensive treatment strategies.

## 5 CONCLUSION

**FIGURE 8:**
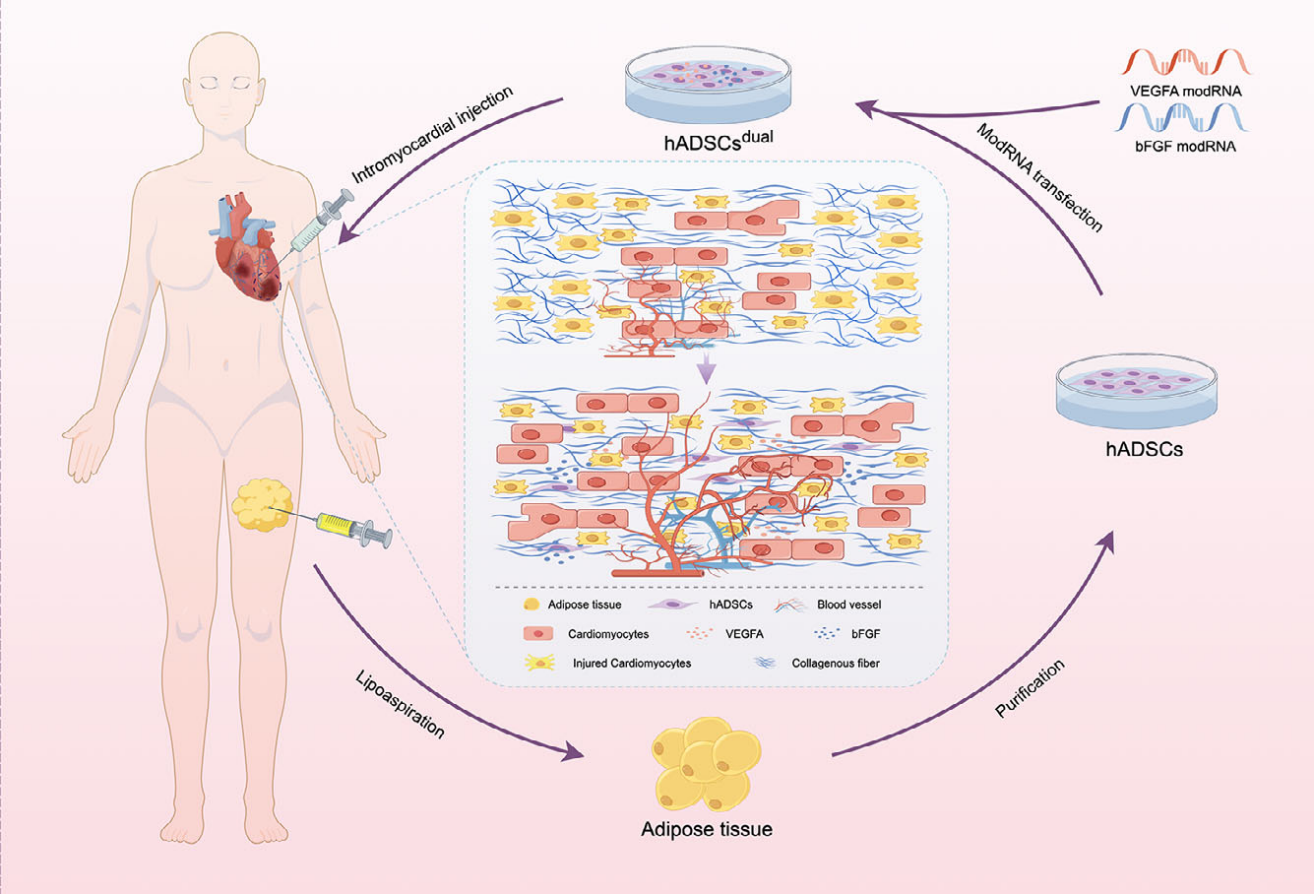
Schematic diagram modelling of cellular engineering and intramyocardial injection delivery of hADSCs enhanced with vascular endothelial growth factor A (VEGFA) and basic fibroblast growth factor (bFGF) modRNA as a novel therapeutic approach for myocardial infarction recovery. Figure created by FigDraw.

In conclusion, our study demonstrates the therapeutic potential of hADSCs overexpressing VEGFA and bFGF in a rat model of acute MI. Specifically, intracardiac injection of these modified hADSCs is able to promote stable vascular regeneration, thus leading to improvements in cardiac function and electrical conduction (Figure 8). This study reports an approach to repaire the damaged myocardium using the combined application of stem cell therapy and mRNA technology. Importantly, this study reports a novel strategy for comprehensively treating MI.

## AUTHOR CONTRIBUTIONS

Wei Wang and Wei Fu conceived the idea. Kaixiang Li, Runjiao Luo, Xindi Yu, Wei Wang and Wei Fu designed the studies. Kaixiang Li and Runjiao Luo performed and analysed the data for most of the experiments. Wei Dong, Guoliang Hao, Dan Hu, Ziyou Yu, Minglu Liu, Tingting Lu, Xiangying Wang, Xin Tang, Xinjun Lin and Huijing Wang assisted with performing experiments and analysing data. Kaixiang Li, Runjiao Luo, Xindi Yu, Wei Wang and Wei Fu wrote the manuscript with the approval of all other authors.

## CONFLICT OF INTEREST STATEMENT

The authors declare no conflict of interest.

### ETHICS STATEMENT

All animal experiments were approved by the Laboratory Animal Welfare Ethics Committee of the Shanghai Children's Medical Center (Approval No, SCMC‐LAWEC‐2019‐009). The acquisition and use of human adipose‐derived stem cells were approved by the Clinical Ethics Committee of Shanghai Ninth People's Hospital (No. SH9H 2018‐T22‐1).

## Supporting information



Supporting Information

## Data Availability

All data are available from the corresponding author uponreasonable request.
