# Supplementary material for: Enhanced human adipose‐derived stem cells with VEGFA and bFGF mRNA promote stable vascular regeneration and improve cardiac function following myocardial infarction
Source: Clin Transl Med. 2025 Feb 26;15(3):e70250. doi: 10.1002/ctm2.70250 (PMC11862888; doi:10.1002/ctm2.70250)
Supplement: Supplementary file 1 — Supporting Information [file CTM2-15-e70250-s001.docx]

**Enhanced human adipose-derived stem cells with VEGFA and bFGF mRNA promote stable vascular regeneration and improve cardiac function following myocardial infarction**

**Supporting Information**

**
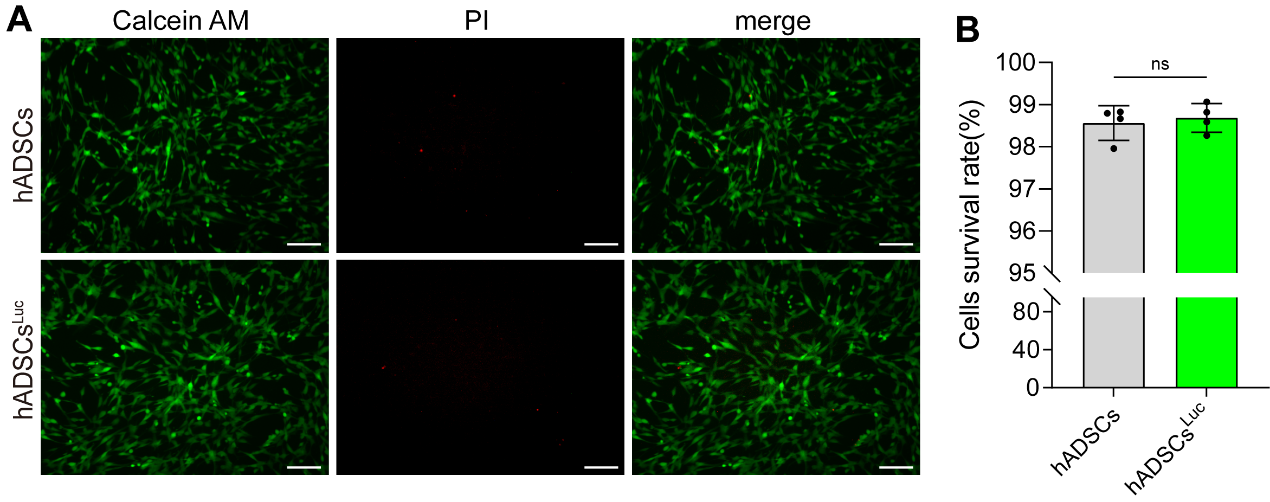
**

**FIGURE S1. Viability of hADSCs following modRNA transfection**

(A) Representative fluorescence images of hADSCs stained with calcein AM and PI 24 h post-transfection with modRNA. Scale bar = 100 μm. (B) Analysis of the survival rates of hADSCs following transfection. ns indicates *p* > 0.05.

**
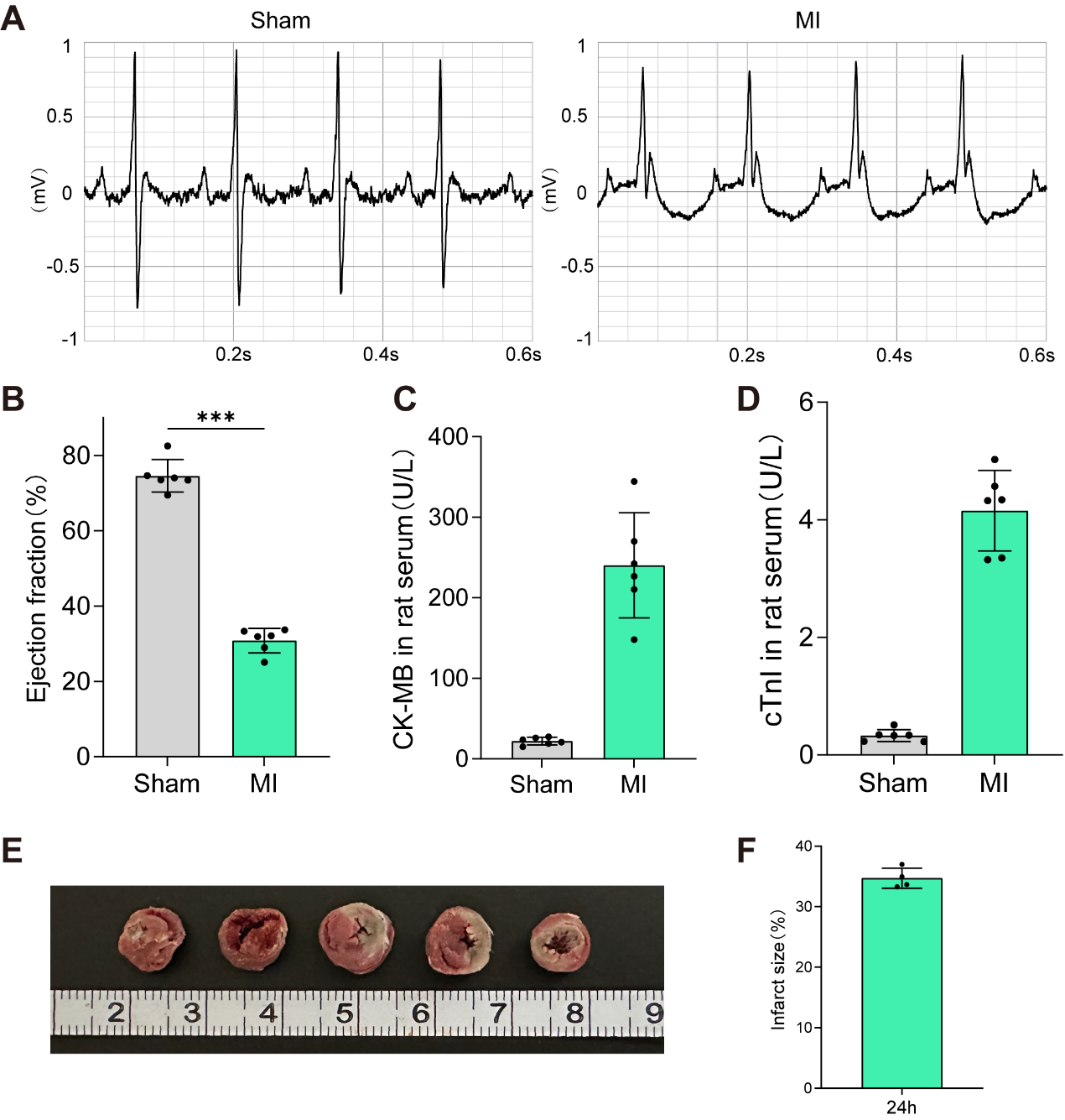
**

**FIGURE S2. Schematic illustration of the MI injury model**

(A) Electrocardiogram changes in rats 5 min after infarction. (B) Ejection fraction of rats after ligation. (C-D) Quantification of CK-MB and cTnI in serum 24 h after MI.  (E) Representative TTC staining demonstrated white ischemic areas with evident blanching, cardiac segments from the base to the apex of the heart are represented from left to right. (F) Quantification of the ratio of ischemic areas 24 hours post-MI.

.


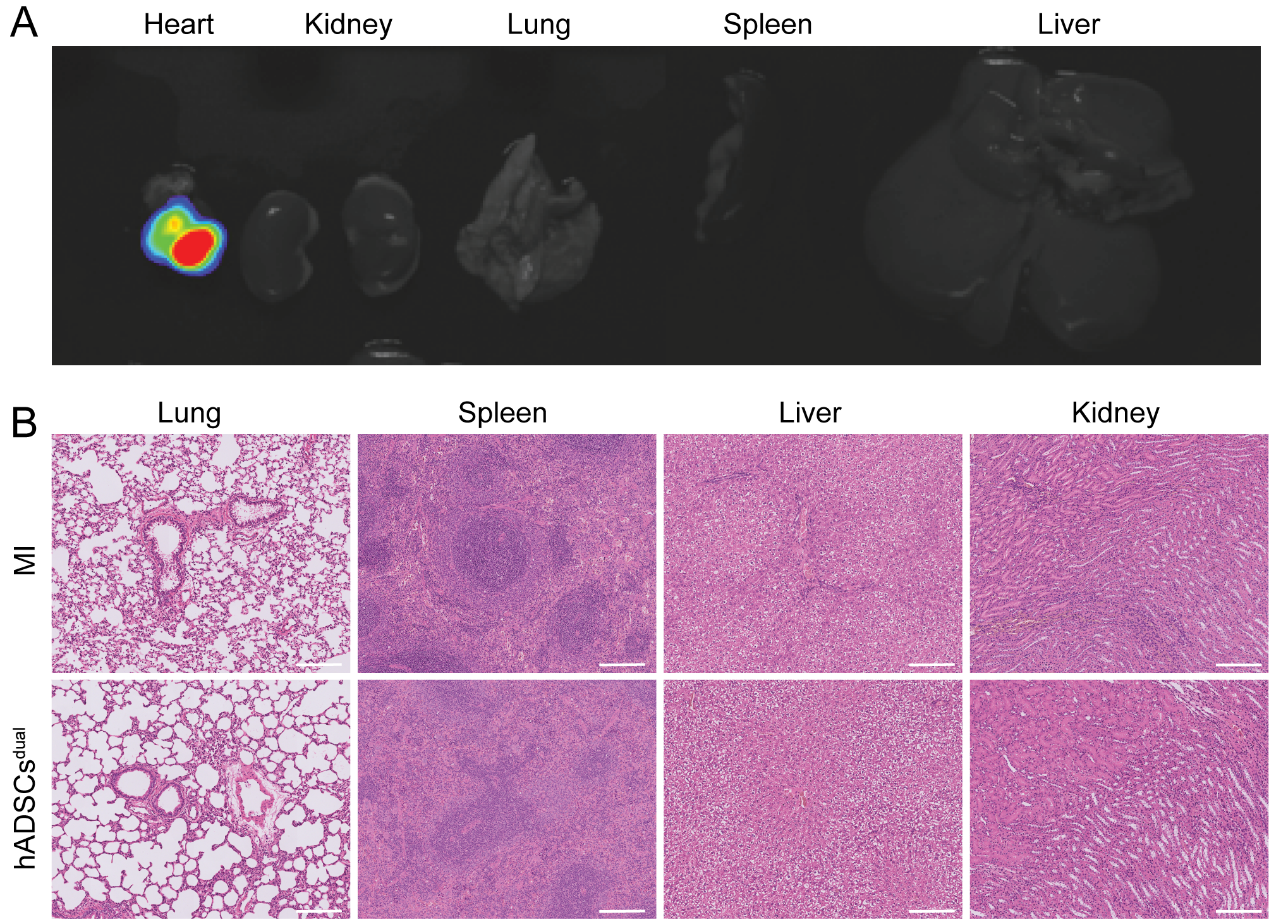


**FIGURE S3. Evaluation of in vivo distribution and safety of hADSCs after transplantation.**
(A) Distribution of hADSCs in vital organs in vivo. (B) Histopathological sections of crucial organs after hADSCs transplantation. Scale bar = 200 μm.

**
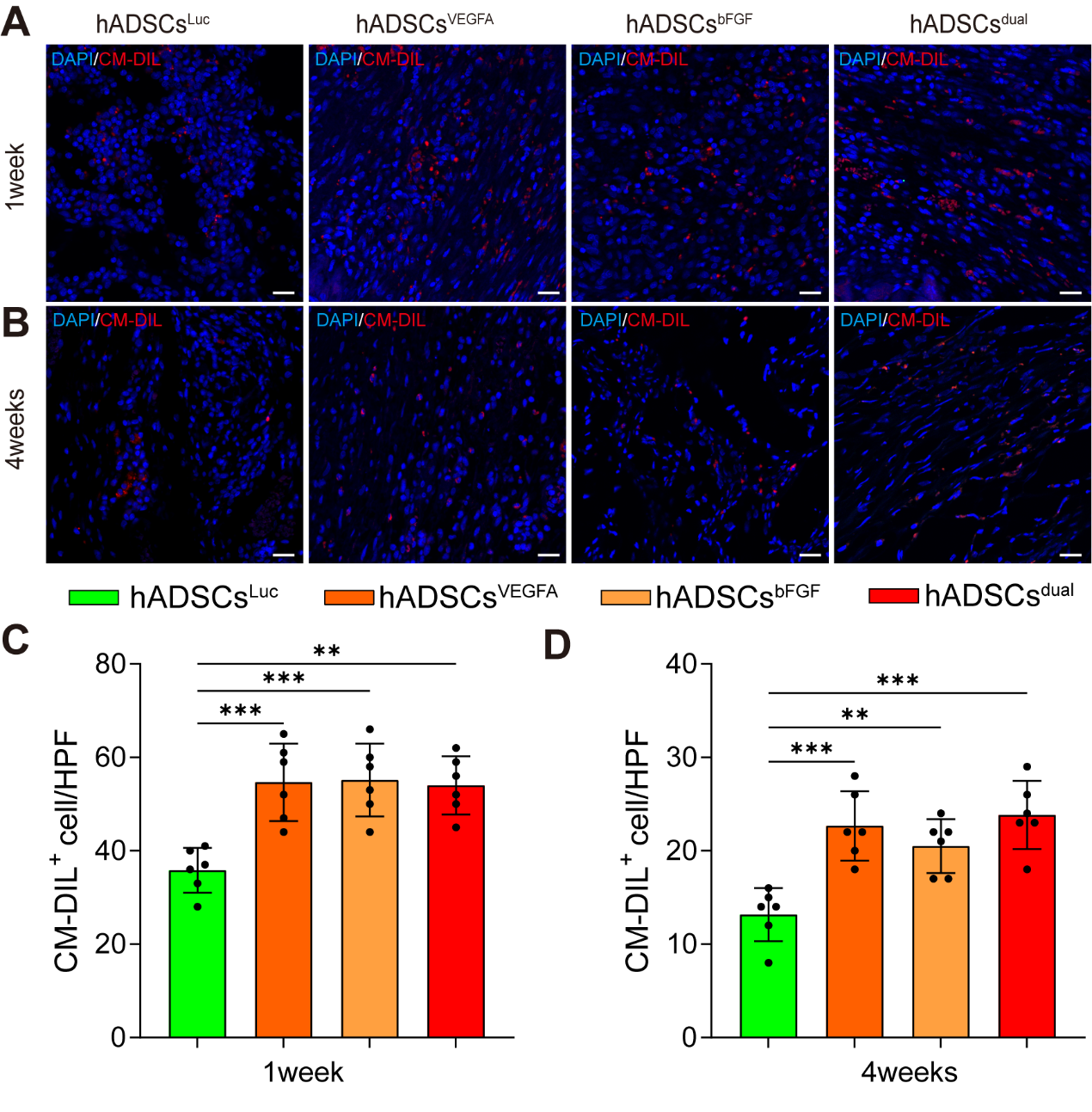
**

**FIGURE S4. Survival of hADSCs at 1 and 4 weeks following MI and treatment**

(A-B) Representative images of hADSCs survival in the infarctional zone at different time points after MI with/without treatment. Scale bar = 25 μm. (C-D) Analysis of the number of survival hADSCs at different time points following MI surgery with/without treatment.


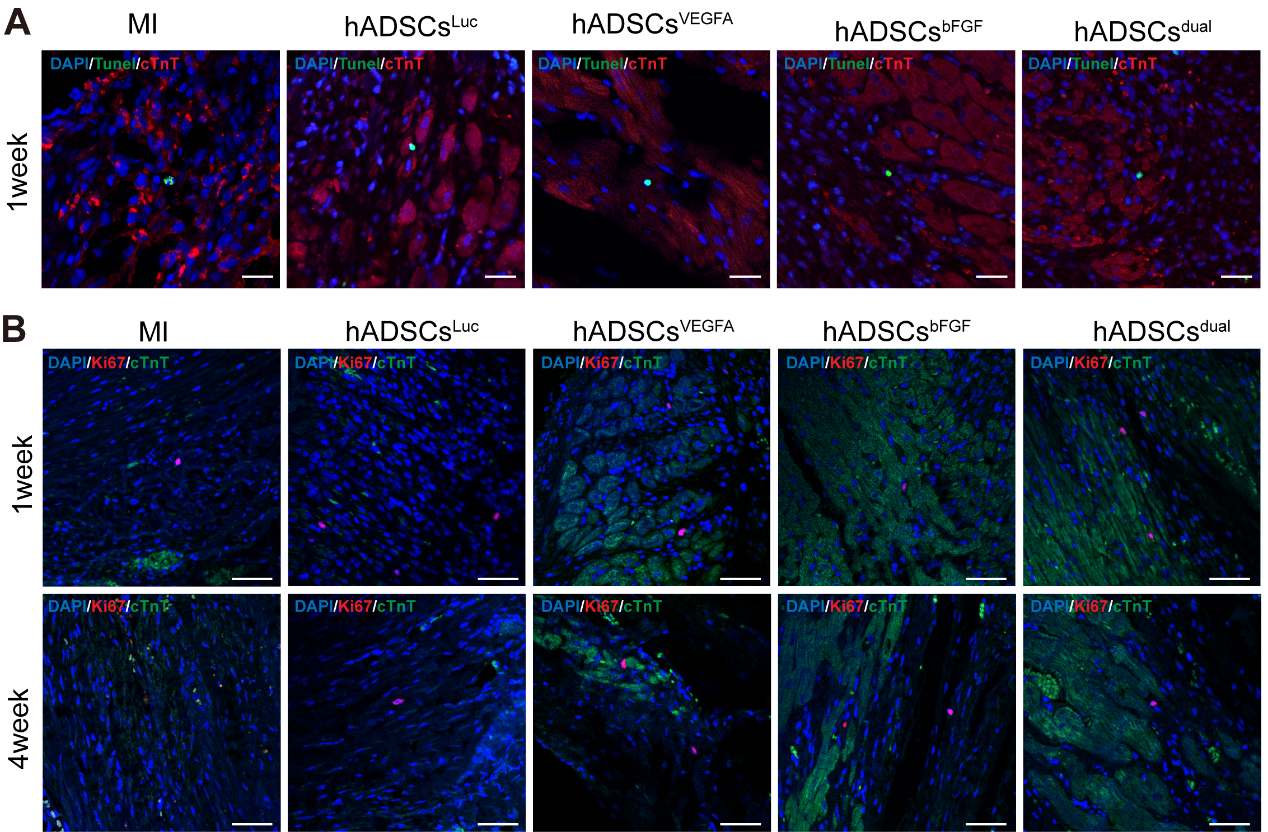


**FIGURE S5.** **Proliferation of cardiomyocytes within the infarction zone.**

(A) Representative tunel and cTnT images 1 week after MI. Scale bar = 25 µm. (B) Representative images of Ki-67 and cTnT at different time points after MI surgery with/without treatment. Scale bar = 100 μm.
